# Responses of plant nutrient resorption to phosphorus addition in freshwater marsh of Northeast China

**DOI:** 10.1038/srep08097

**Published:** 2015-01-29

**Authors:** Rong Mao, De-Hui Zeng, Xin-Hou Zhang, Chang-Chun Song

**Affiliations:** 1 Key Laboratory of Wetland Ecology and Environment, Northeast Institute of Geography and Agroecology, Chinese Academy of Sciences, Changchun 130102, China; 2 State Key Laboratory of Forest and Soil Ecology, Institute of Applied Ecology, Chinese Academy of Sciences, Shenyang 110016, China

## Abstract

Anthropogenic activities have increased phosphorus (P) inputs to most aquatic and terrestrial ecosystems. However, the relationship between plant nutrient resorption and P availability is still unclear, and much less is known about the underlying mechanisms. Here, we used a multi-level P addition experiment (0, 1.2, 4.8, and 9.6 g P m^−2^ year^−1^) to assess the effect of P enrichment on nutrient resorption at plant organ, species, and community levels in a freshwater marsh of Northeast China. The response of nutrient resorption to P addition generally did not vary with addition rates. Moreover, nutrient resorption exhibited similar responses to P addition across the three hierarchical levels. Specifically, P addition decreased nitrogen (N) resorption proficiency, P resorption efficiency and proficiency, but did not impact N resorption efficiency. In addition, P resorption efficiency and proficiency were linearly related to the ratio of inorganic P to organic P and organic P fraction in mature plant organs, respectively. Our findings suggest that the allocation pattern of plant P between inorganic and organic P fractions is an underlying mechanism controlling P resorption processes, and that P enrichment could strongly influence plant-mediated biogeochemical cycles through altered nutrient resorption in the freshwater wetlands of Northeast China.

In recent decades, human activities have drastically increased phosphorus (P) inputs to the Earth's surface through fertilizer use, recycling of crop residues and manures, and discharges of urban and industrial wastes^1^,^2^. It has been estimated that anthropogenic P inputs have doubled the natural P loading, which has led to widespread eutrophication of terrestrial, freshwater, and marine ecosystems^3^,^4^. Since P is one of the most important nutrients limiting primary producers in most ecosystems^4^,^5^, increased P loading has the potential to substantially influence plant growth and nutrient status in plant organs, affecting ecosystem carbon and nutrient cycles. Despite the fact that P enrichment is widespread all over the world^2^, the impacts of increased P inputs on biogeochemical cycles of carbon and nutrients are not well understood.

Nutrient resorption from senescing plant organs is an important aspect of plant internal nutrient cycle that reduces plant dependence on external nutrient availability, and thus is a key strategy for nutrient conservation in plants and contributes to plant nutrient use efficiency^6^,^7^,^8^. Meanwhile, nutrient resorption controls litter decomposition and nutrient release via its effect on litter quality, which in turn produces a positive feedback to soil nutrient availability and affects ecosystem productivity and elemental cycling^9^,^10^. Increased P loading could directly affect soil nutrient availability and thus nutrient resorption pattern^11^.

Phosphorus enrichment generally increases P concentration in mature leaves and reduces plant P resorption proficiency (i.e. the level to which nutrient concentration is reduced in senesced organs^7^) in fertilization studies^12^,^13^,^14^. Meanwhile, plant P resorption efficiency (i.e. the proportion of nutrient that is resorbed during senescence^15^) is unaffected^14^,^16^,^17^ or decreased^18^,^19^,^20^ by P fertilization. However, the mechanism of the response of plant P resorption to P enrichment is still unclear. Recently, Hidaka and Kitayama^21^ have suggested that allocation of P fractions in leaves can determine the variation of P resorption with different soil P availabilities in tropical montane rain forests. To date, relatively few studies have examined the response of P fractions (i.e. inorganic vs. organic P) in plants to increased P loading^14^,^22^. Among the P fractions, inorganic P fraction is highly mobile and can be easily withdrawn during senescence, whereas organic P fraction must be hydrolyzed before it can be resorbed^14^,^15^. Therefore, knowledge about the allocation patterns of P fractions in plants is needed to unravel the underlying mechanism controlling the response of plant P resorption to increased P inputs.

In contrast to P resorption, the response of plant nitrogen (N) resorption to P fertilization is much more complex^23^. Some studies have shown that increased P loading decreases N concentration in mature leaves and increases N resorption efficiency and proficiency^13^,^19^, whereas several other studies have observed unchanged mature and senesced leaf N concentrations, and thus N resorption efficiency following P addition^17^,^18^,^20^. In addition, Mayor et al.^14^ have found that, despite unaltered mature leaf N concentration in a lowland tropical forest, long-term P fertilization produces neutral or negative effects on N resorption efficiency and proficiency for different tree species. These inconsistent results may be caused by the species-specific response, the differences in P input rates, and the type of nutrient limitation^23^. Explorations of inter-specific variations in N resorption patterns with different P addition rates would help clarify how N resorption responds to P enrichment.

Although the effects of P enrichment on leaf nutrient resorption have been widely studied^14^,^16^,^17^, data on non-leaf organs remain extremely limited. There is a particular need to address knowledge gap on how non-leaf organ nutrient resorption responds to altered P availability, given the high nutrient resorption capacity of non-leaf organs^24^ and the different response patterns of leaf and non-leaf organs to nutrient addition^25^. Moreover, little is known about how change in P availability affects nutrient resorption at both species and community levels. Increased P inputs can not only alter biomass allocation pattern between leaf and non-leaf organs within individual species^13^, but also cause substantial changes in vegetation community structure^26^,^27^. Altered vegetation community structure and biomass allocation pattern would drive the changes in species- and community-level plant resorption. In order to better understand the relationship between soil P availability and plant nutrient resorption, we need to investigate the effects of P addition on nutrient resorption at different hierarchical levels.

Here, we conducted a multi-level P fertilization experiment to identify the responses of plant organ-, species-, and community-level nutrient resorption to P enrichment in a freshwater marsh in the Sanjiang Plain, Northeast China. The specific objectives of our study are to answer the following three questions: first, how do leaf and stem N resorption efficiency and proficiency vary across different P addition levels within individual species? Second, how does multi-level P addition affect leaf and stem P resorption efficiency and proficiency? Is allocation of plant P fractions the major mechanism controlling P enrichment-induced changes in P resorption patterns? Third, how do species- and community-level nutrient resorptions respond to increased P availability? Is the community-level response driven by the altered vegetation composition and structure, or by the plasticity of individual species?

## Results

### Effects of P addition on aboveground plant biomass

*D.* *angustifolia* leaf and stem biomass generally declined, while *G.* *spiculosa* leaf and stem biomass increased with increasing P addition levels (Table 1). In total, total aboveground biomass of the two wetland species increased with increasing P addition rates (Table 1).

### Effects of P addition on nutrient resorption at the plant organ level

**Figure 1:**
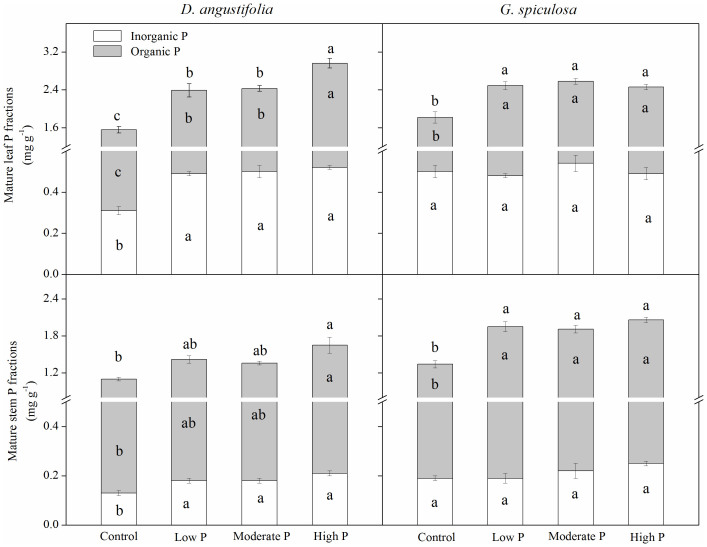
Effect of P addition on P fractions in mature plant organs. Different lowercase letters in the bars and above the bars indicated significant differences in P fractions and total P concentration among the four treatments, respectively.

For both *D.* *angustifolia* and *G.* *spiculosa*, P addition increased mature leaf and stem N and P concentrations (Appendix Table S1 and Figure 1). However, P addition and plant species produced a significant interaction on inorganic and organic P fractions in mature plant organs (Appendix Table S2). Phosphorus addition caused an increase in organic P fraction in mature leaves and stems for the two wetland species (Figure 1). However, P addition only increased inorganic P concentration in *D.* *angustifolia* leaf and stem, but did not affect inorganic P concentration in *G.* *spiculosa* leaf and stem (Figure 1).

**Figure 2:**
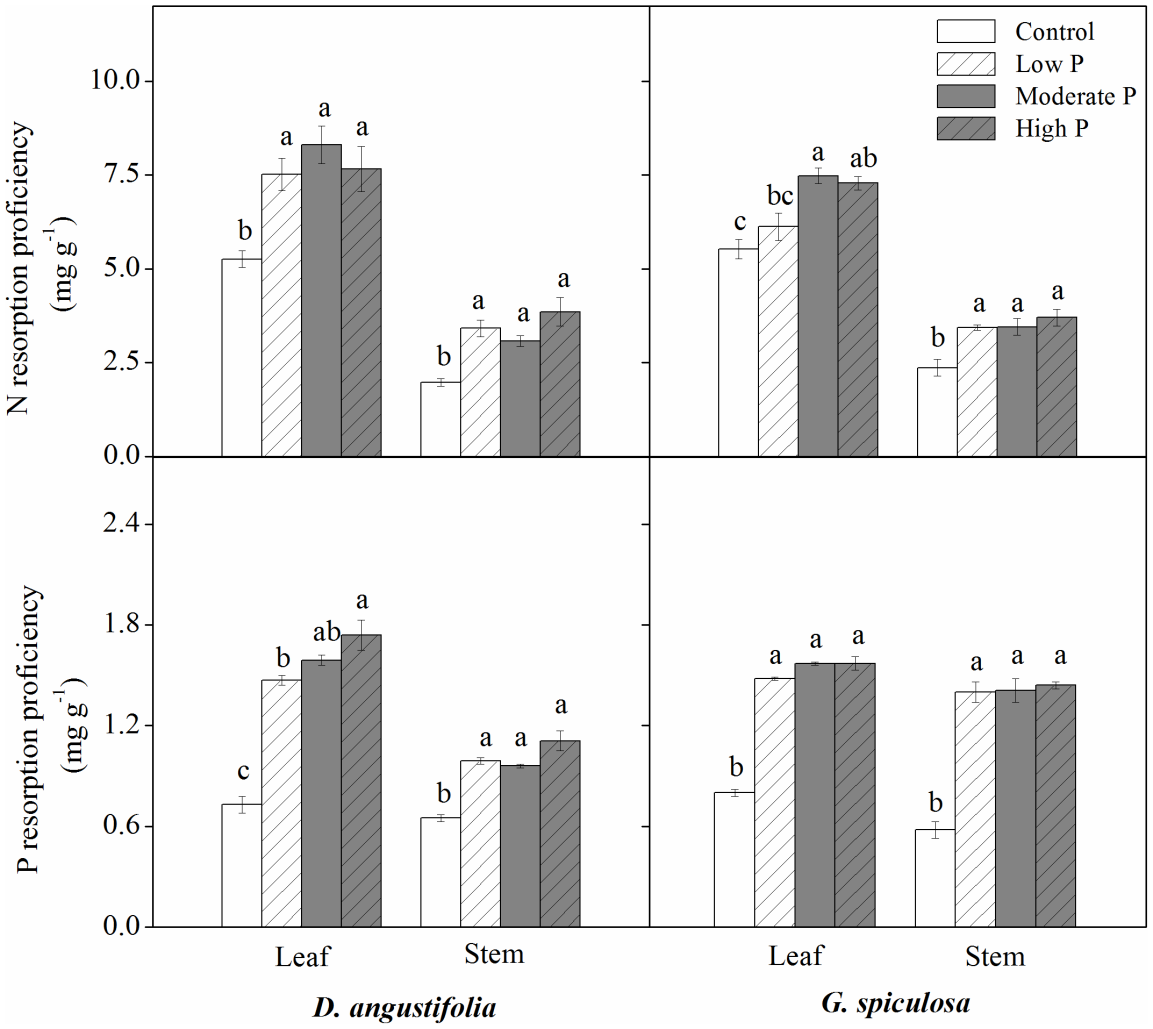
Effect of P addition on nutrient resorption proficiency of plant organs. Different lowercase letters indicated significant differences among the four treatments.

**Figure 3:**
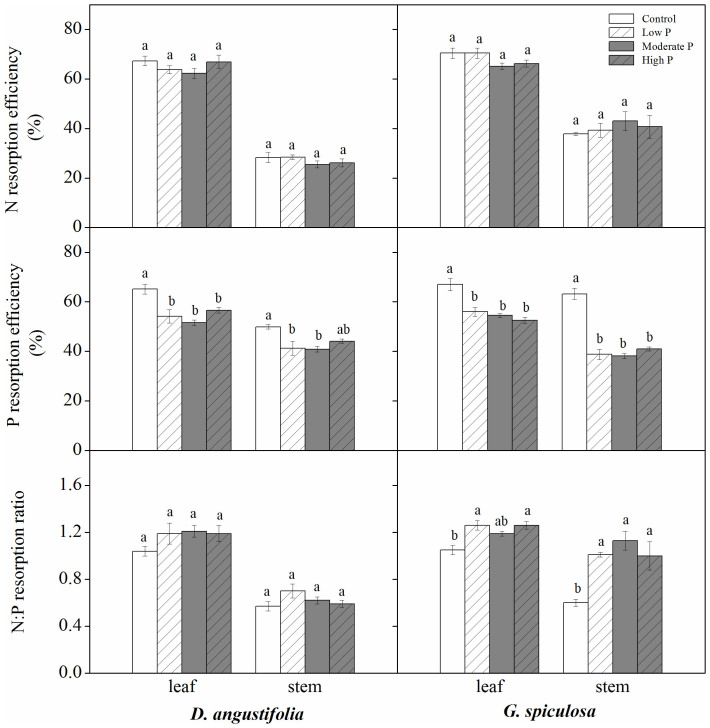
Effect of P addition on nutrient resorption efficiency of plant organs. Different lowercase letters indicated significant differences among the four treatments.

For both leaf and stem of the two wetland plants, P addition caused decreases in N and P resorption proficiency (Figure 2). Phosphorus addition did not affect N resoprtion efficiency, but decreased P resorption efficiency (Figure 3). Generally, there was no significant difference in N and P resorption proficiency, and N and P resorption efficiency among low P, moderate P and high P treatments (Figures 2 and 3). In addition, P addition did not impact N:P resorption ratio of *D.* *angustifolia* leaf and stem, but caused an increase in N:P resorption ratio of *G.* *spiculosa* leaf and stem (Figure 3).

**Figure 4:**
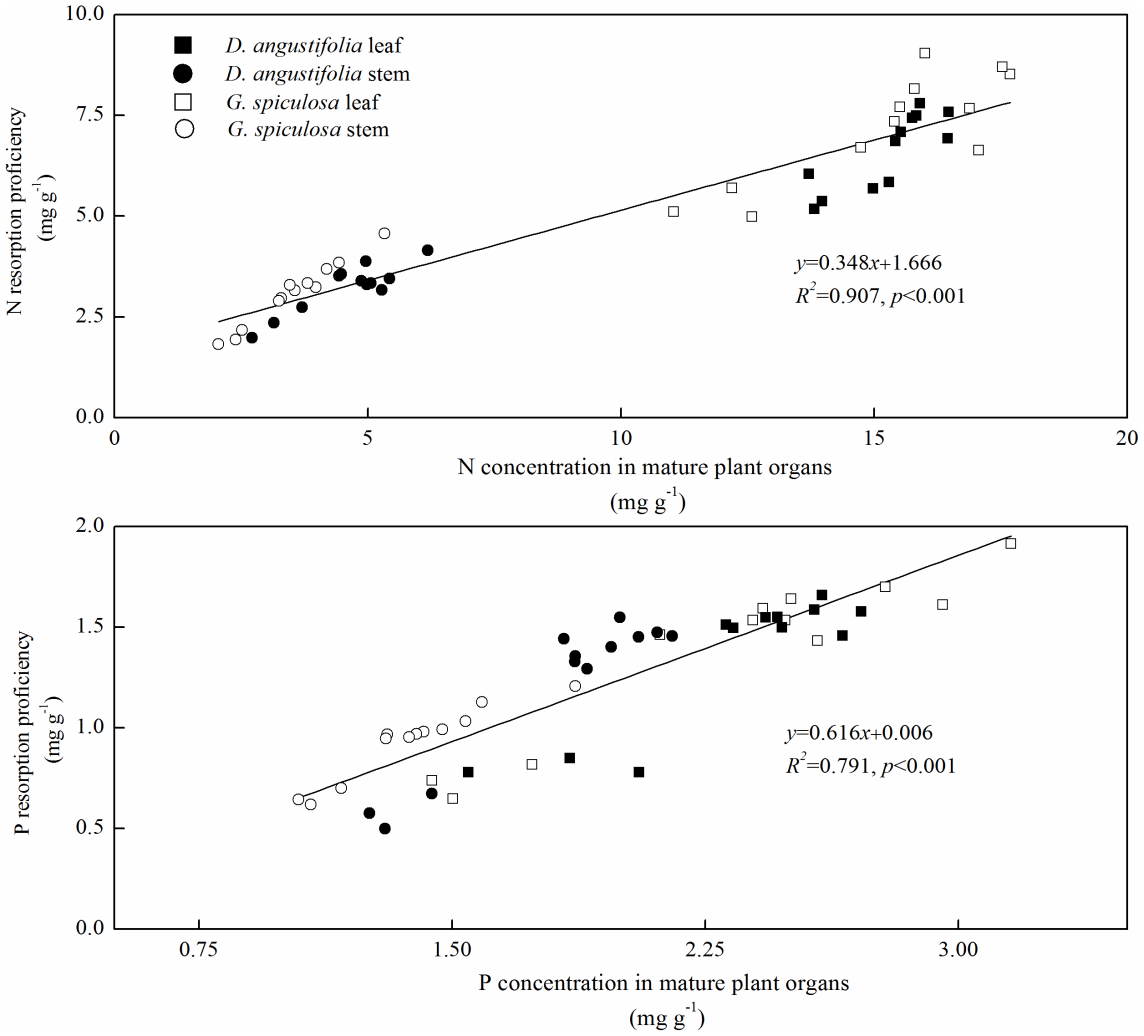
Relationship of nutrient concentrations in mature plant organs and nutrient resorption proficiency.

**Figure 5:**
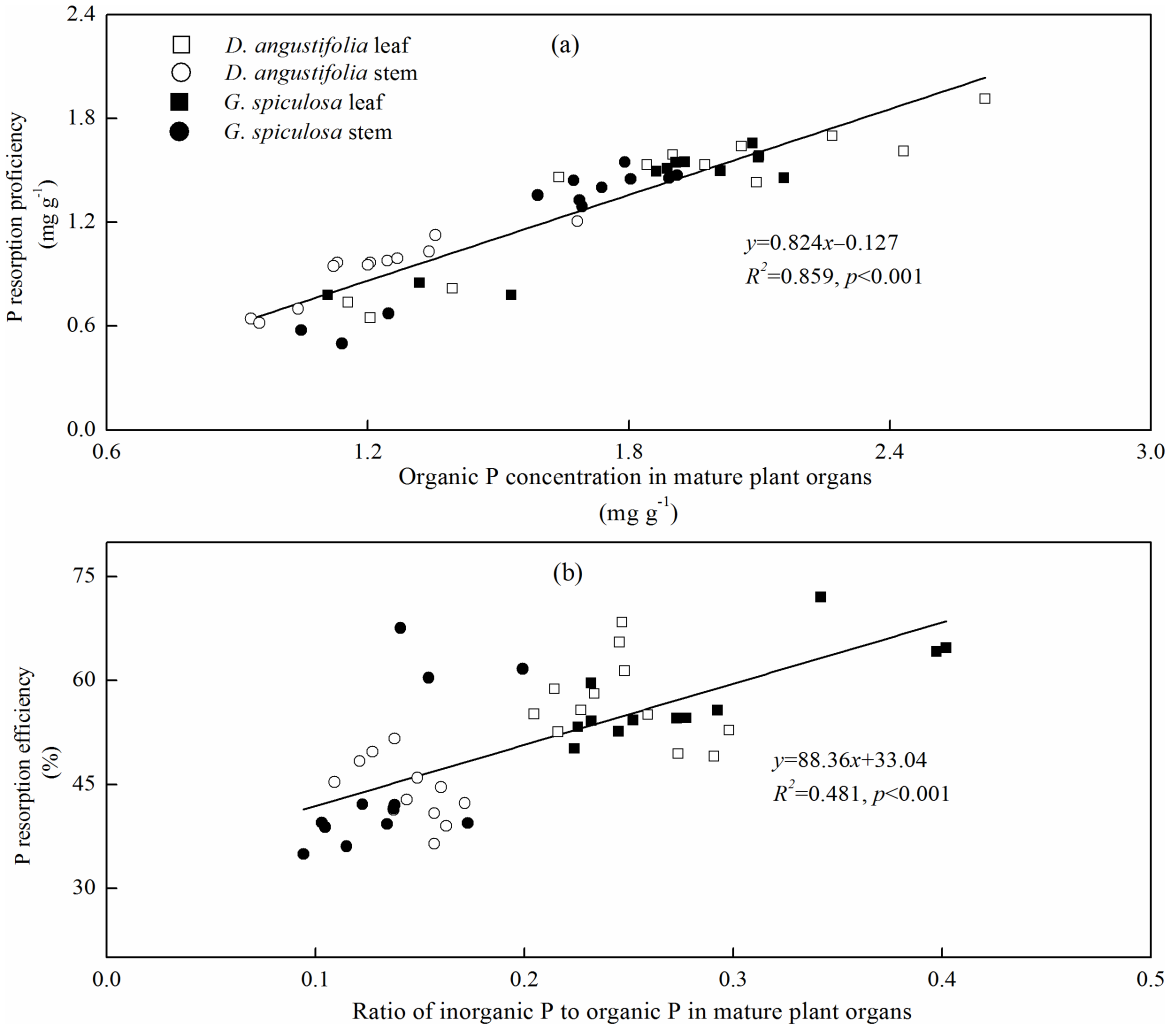
Relationships (a) between P resorption proficiency and organic P fraction in mature plant organs, and (b) between P resorption efficiency and the ratio of inorganic P to organic P in mature plant organs.

Nitrogen and P resorption proficiency showed a significant linear relationship with N and P concentrations in mature plant organs, respectively (Figure 4). Phosphorus resorption proficiency also linearly correlated with organic P concentration in mature plant organs (Figure 5a). In addition, there was a positive relationship between P resorption efficiency and the ratio of inorganic P to organic P in mature plant organs (Figure 5b).

### Effects of P addition on plant nutrient resorption at the species level

At the species level, P addition did not change N resorption efficiency, but decreased N resorption proficiency for both species (Table 2). In addition, P addition caused declines in species-level P resorption efficiency and proficiency for both species (Table 2).

### Effects of P addition on plant nutrient resorption at the community level

At the community level, plant N and P resorption proficiency decreased with increasing P addition rates (Table 3). However, P addition did not affect community-level N resorption efficiency, but decreased community-level P resorption efficiency (Table 3). In addition, P addition increased N:P resorption ratio at the community level (Table 3).

## Discussion

In this study, adding P with different levels generally had the similar effects on plant nutrient resorption parameters across the three hierarchical levels. These findings provided evidence that there existed a threshold for P enrichment-induced effects on plant nutrient resorption. Moreover, the critical threshold was lower than 1.2 g P m^−2^ year^−1^ in this freshwater wetland.

Surprisingly, P addition decreased both leaf and stem N resorption proficiency of the two wetland plants, which is contrary to the previous studies showing that P enrichment produced positive or neutral effects on N resorption proficiency^13^,^17^,^20^. Generally, N resorption proficiency depends on mature plant organ N status^10^,^11^. In this study, N resorption proficiency was negatively correlated with N concentration in mature plant organs (Figure 4). In this freshwater marsh, increased P inputs probably enhanced the ability of plant N acquisition to maintain a balanced N:P stoichiometry^28^, resulting in a marked increase in plant N concentration (Appendix Table S1) and a decline in N resorption proficiency. Generally, excessive P addition will shift it to N limitation of plant growth in terrestrial and aquatic ecosystems^5^. Our result suggests that, in the context of P enrichment, plasticity in N resorption proficiency could increase the amount of N returned to the soils and produce a positive feedback to soil N availability^29^, which may alleviate the enhanced N limitation and impact on plant nutrient economics.

Our result showed that N resorption efficiency of the two plant species remained unchanged following 6 years of P addition, although there was a substantial increase in mature plant organ N concentration. In previous fertilization studies, the relationship between N resorption efficiency and mature leaf N concentration was still elusive^14^,^17^,^25^. In the present study, we did not find a clear relationship between N resorption efficiency and mature plant organ N concentration (data not shown) either. Generally, N resorption process is not only controlled by plant N concentration, but also by the allocation patterns between N fractions (e.g. soluble and insoluble fractions) in plant organs^30^. Despite an increase in mature plant organ N concentration, increased P loading might not alter N allocation patterns in mature plant organs^22^, and hence did not affect N resorption efficiency in this freshwater marsh. In order to unravel the mechanisms controlling the response of N resorption efficiency to changing P availability, further studies are needed to evaluate the effect of P addition on N fractions in mature plant organs.

In the present study, 6 years of P addition decreased plant organ P resorption proficiency, which is in agreement with the previous studies^13^,^14^. Increased mature plant organ P concentration following P addition contributed to the reduction in P resorption proficiency^10^,^11^. In this study, mature plant P concentration accounted for about 79% of the variation observed in plant P resorption proficiency (Figure 4). Moreover, we found that organic P fraction in mature plant organs contributed to approximately 86% of the variation in plant P resorption proficiency (Figure 5). Generally, organic P fraction is more immobile than inorganic P fraction during the process of resorption^14^,^21^. Therefore, decreased plant P resorption proficiency following P addition may be attributed to the increase in organic P fraction in mature plants.

In line with some previous studies^19^,^20^, we also observed that P addition caused a decline in P resorption efficiency. However, some other studies showed that plant P resorption efficiency was not affected by increased P availability in Neotropical savanna^16^, temperate peatland^17^ and lowland tropical forest^14^. These inconsistent responses of P resorption efficiency to P addition may be explained by the different allocation patterns between organic and inorganic P fractions in mature plant organs. Mayor et al.^14^ found that 13 years of P addition resulted in similar increases in foliar organic and inorganic P fractions, and thus did not affect P resorption efficiency in a lowland tropical forest. In contrast, 6 years of P addition generally increased the proportion of organic P fraction in mature plant organs in this study (Figure 1). Meanwhile, P resorption efficiency exhibited a positive linear relationship with the ratio of inorganic P to organic P in mature plant organs (Figure 5b). These results suggest that the allocation pattern of P between inorganic and organic P fractions would be a simple and powerful indicator of plant P resorption efficiency.

We observed that the effects of P addition on plant N and P resorption were similar across the three hierarchical levels. At both species and community levels, P addition caused decreases in N resorption proficiency, P resorption proficiency and efficiency, and had no effect on N resorption efficiency (Tables 2 and 3). These similar responses were caused by the consistent responses of leaf and stem N and P resorption in different species to increased P availability. Although nutrient resorption at plant organ, species, and community levels exhibited identical responses to P addition, increased P input caused a shift in vegetation community composition and altered biomass allocation between leaves and stems (Table 1). Considering that N and P resorption parameters varied with species and plant organs, knowledge about the community-level nutrient resorption helps to accurately assess plant internal nutrient cycles in the context of P enrichment in the freshwater marsh.

Recently, Reed et al.^9^ observed a notable increase in N:P resorption ratio with increasing absolute latitudes, and suggested that variations in N:P resorption stoichiometry may provide a complementary metric to assess the variations of nutrient cycles and limitation across environmental gradients. In this study, community-level N:P resorption ratio was <1 in the control treatments, and was >1 in the P addition treatments (Table 2). Moreover, P addition resulted in an increase in aboveground plant biomass (Table 1). These results indicate that this freshwater marsh is P limitation or N and P co-limitation^5^,^9^, and imply that excessive P loading would cause this freshwater marsh become an N-limited ecosystem. Consequently, *G.* *spiculosa* gained an advantage over *D.* *angustifolia* following P addition in this ecosystem (Table 1), because of the high N resorption efficiency^31^.

In the present study, the response of N:P resorption ratio to P addition differed with species (Figure 3). These differential responses may reflect the difference in genetic adaption of P resorption to P availability between the two dominant species^23^, given the unchanged N resorption efficiency. Compared with *D.* *angustifolia*, P addition only increased organic P fraction in mature organs of *G.* *spiculosa*. Therefore, the magnitude of the decline in P resorption efficiency of *G.* *spiculosa* was greater than that of *D.* *angustifolia* (Figure 3). Following 6 years of P addition, *G.* *spiculosa* leaf and stem N:P resorption ratios declined, but *D.* *angustifolia* leaf and stem N:P resorption ratios remained unchanged.

In summary, we examined the changes in N and P resorption across the three hierarchical levels (plant organ, species, and community) following 6 years of P addition in a freshwater marsh, Northeast China. We observed that, for the two dominant species (*D.* *angustifolia* and *G.* *spiculosa*), leaf and stem nutrient resorption responded consistently to P addition. Specifically, P addition exerted a negative effect on N resorption proficiency, P resorption efficiency and proficiency, but did not affect N resorption efficiency. Consequently, the responses of species- and community-level nutrient resorption to P addition were similar to those at the plant organ level. Moreover, P resorption efficiency and proficiency are both tightly correlated with the ratio of inorganic P to organic P and organic P fraction in plant mature organs. Our results suggest that nutrient resorption at different hierarchical levels exhibits similar responses to P addition, and that the allocation patterns of P between inorganic and organic P fractions in plant mature organs could effectively indicate the variations of P resorption in the context of P enrichment. Given the widespread occurrence of increased P loading^3^,^4^, our study could provide insights into plant-mediated biogeochemical cycles in temperate freshwater wetlands of Northeast China.

## Methods

### Study site and experiment design

This study was conducted in a freshwater marsh at the Sanjiang Mire Wetland Experimental Station (47°35′N, 133°31′E and 56 m a.s.l.), which is located in the Sanjiang Plain, Heilongjiang Province, Northeast China. The Sanjiang Plain is one of the largest freshwater marshes in China, and the area of marshes was approximately 8.10 × 10^5^ ha in 2005^32^. The study site is characterized by temperate continental monsoon climate with a mean annual (1990–2010) precipitation of 566 mm and a mean annual temperature of 2.5°C. Soil pH value is 5.35, and organic C, total N, and total P concentrations at 0–15 cm depth are 95, 6.8, and 1.45 mg g^−1^, respectively. According to the long-term monitoring data of the Sanjiang Mire Wetland Experimental Station, the amount of P input to marshes during the growing season was about 0.4 g P m^−2^ year^−1^. The increasing P input in the freshwater marshes in the Sanjiang Plain received is mainly due to the fertilizer application during agricultural activities.

In this study, we established a P addition experiment in the autumn of 2006. To simulate the future possible increases in P input to this marsh, we experimentally increased the ambient growing season P loading by factors of 3, 12, and 24. There were four P addition treatments (control, 0 g P m^−2^ year^−1^; low P, 1.2 g P m^−2^ year^−1^; moderate P, 4.8 g P m^−2^ year^−1^; and high P, 9.6 g P m^−2^ year^−1^) with triplicates for each treatment. A total of 12 plots, each of 1 m × 1 m, were arranged in a complete randomized block design and separated by a 1-m buffer zone. Each plot was fenced with plastic (PVC) frames (1 m × 1 m, 0.5 m in depth) to prevent horizontal movement and lateral loss of the added P. Meanwhile, board walks giving access to the whole experimental area were installed to minimize further disturbance on the plots. The P addition experiment was initiated in 2007, and P was applied as NaH_2_PO_4_ solution every two weeks from early May to late August each year.

### Field sampling and measurement

All plants in two quadrats (0.3 m × 0.3 m) were harvested to measure aboveground biomass within each plot in early August 2012. Plant biomass was separated into leaf and stem for each species, over-dried at 65°C to constant weight, and then weighed. In all plots, the dominant species were *D.* *angustifolia* and *G.* *spiculosa* that accounted for more than 95% of the total aboveground biomass, and the accompanying species were *Galium boreale*, *Lycopus lucidus*, *Gentiana manshurica*, and *Stellaria longifolia*. Thus, we only measured total aboveground plant biomass and nutrient resorption of the two dominant species in these plots.

In each plot, thirty shoots with similar height were randomly selected for *D.* *angustifolia* and *G.* *spiculosa* in early August 2012, respectively. Fifteen shoots were sampled to determine nutrient concentrations of mature leaf (including blade and sheath) and stem. We collected two fully expanded leaves per shoot, and one stem per shoot. Meanwhile, we marked another fifteen shoots with plastic labels, and tagged two fully expanded leaves per shoot by tying a small tag to the leaf base. Censuses of the labeled organs were performed every three days from late September to early October in 2012, and the labeled organs were considered senesced when they thoroughly turned yellow or brown. Senesced organs were harvested in the same way as the mature organs. Both mature and senesced plant organs were oven-dried at 65°C to constant weight and weighed separately, and the average masses of individual leaf and stem were calculated. Plant samples were ground and stored for chemical analysis.

For each plant sample, 0.1 g subsample was digested with concentrated H_2_SO_4_, and then N and P concentrations in the digests were determined by the sodium salicylate-sodium hypochlorite colorimetric method and the molybdenum blue colorimetric method using a continuous-flow autoanalyzer (AutoAnalyzer III, Bran + Luebbe GmbH, Germany), respectively. Mature leaf and stem inorganic P concentration was measured according to the method of Thomas et al.^33^. Twenty milligram plant material was extracted with 1% HClO_4_ at room temperature for one hour, and inorganic P in the extracts was determined by the molybdenum blue colorimetric method^34^. Organic P concentration in the plant samples was calculated as the difference between total P and inorganic P.

Given the mass loss during plant organ senescence, nutrient resorption efficiency was calculated as the ratio of the difference in nutrient pool between mature and senesced organs to nutrient pool in mature plant organ^35^. Total nutrient pool of individual organ (for both mature and senesced materials) was calculated by multiplying the mass of individual organ with its nutrient concentration. Meanwhile, nutrient resorption proficiency was defined as the nutrient concentration in senesced material; low nutrient concentration in senesced material corresponded to high nutrient proficiency^7^. In each plot, species-level nutrient resorption parameters were assessed using the relative biomass of each plant organ and its nutrient resorption parameters within individual species^25^.

In this study, community-level nutrient resorption parameters were calculated using biomass-weighted means of two dominant species presented in the plot^27^. Community-level plant nutrient pool was obtained based on the aboveground biomass and nutrient concentration of the two dominant species. At the community level, mature or senesced plant nutrient concentration was obtained by dividing plant nutrient pool by the corresponding plant biomass in each plot. Community-level nutrient resorption efficiency was calculated as the percentage reduction in nutrient pool between mature and senesced plants in the community.

### Statistical analyses

All statistical analyses were performed with SPSS 13.0 for Windows (SPSS Inc. 2004) and the accepted significance level was *α* *= * 0.05. Before statistical analysis, the normality of data was tested by Levene's test, and all non-normal data were transformed to follow a normal distribution. Data of senesced N and P concentrations were log_10_(*x*)-transformed, and N and P resorption efficiencies were arcsine(*x*) transformed. Three-way analysis of variance (ANOVA) was used to examine the effects of P addition levels, species, plant organs and their interactions on nutrient resorption parameters. One-way ANOVA was used to test the effect of P addition levels on community-level nutrient resorption parameters. Tukey's honest significant difference test was used to determine the significant difference in nutrient resorption parameters among all treatments. Linear regression analyses were used to examine the relationships between nutrient (N and P) concentration in mature organs and nutrient resorption proficiency, between P resorption proficiency and organic P fraction in mature plant organs, and between P resorption efficiency and the ratio of inorganic P to organic P in mature plant organs.

## Author Contributions

R.M. and C.C.S. designed the study, R.M. and D.H.Z. conducted analyses and wrote the paper, and X.H.Z. discussed the draft manuscript and interpreted the results.

## Supplementary Material

Supplementary Information Responses of plant nutrient resorption to phosphorus addition in freshwater marsh of Northeast China

## Figures and Tables

**Table 1:** Effect of P addition on aboveground plant biomass

Plant biomass (g m^−2^)	Control	Low P	Moderate P	High P	*P*-values
*D. angustifolia* leaf	84.2(7.6)a	66.3(1.9)ab	52.1(5.5)b	22.1(5.8)c	<0.001
*D. angustifolia* stem	75.8(7.0)a	59.6(2.2)a	51.7(5.8)a	20.4(7.3)b	0.001
*G. spiculosa* leaf	57.9(7.9)c	100.8(5.1)bc	127.5(17.1)b	220.0(10.8)a	<0.001
*G. spiculosa* stem	32.9(4.8)c	61.3(2.9)bc	73.8(10.1)b	102.9(5.2)a	<0.001
Total aboveground biomass	250.8(26.3)b	287.9(9.7)ab	305.0(27.6)ab	365.4(9.1)a	0.023

Note: Data in parentheses are the standard errors of means (*n* = 3). Different lowercase letters in the same rows indicated significant differences among the four treatments.

**Table 2:** Effect of P addition on plant nutrient resorption at the species level

	Control	Low P	Moderate P	High P	*P*-values
N resorption proficiency (mg g^−1^)					
*D. angustifolia*	3.70(0.09)b	5.58(0.26)a	5.69(0.23)a	5.87(0.41)a	0.002
*G. spiculosa*	4.38(0.24)c	5.11(0.22)bc	6.00(0.23)ab	6.14(0.16)a	0.001
P resorption proficiency (mg g^−1^)					
*D. angustifolia*	0.70(0.04)c	1.25(0.01)b	1.27(0.03)b	1.45(0.02)a	<0.001
*G. spiculosa*	0.72(0.03)b	1.45(0.03)a	1.51(0.03)a	1.53(0.03)a	<0.001
N resorption efficiency (%)					
*D. angustifolia*	48.8(1.9)	47.0(1.1)	44.1(1.1)	48.1(1.7)	0.210
*G. spiculosa*	58.6(1.5)	58.6(1.4)	57.0(1.8)	58.0(2.6)	0.921
P resorption efficiency (%)					
*D. angustifolia*	57.9(1.6)a	48.0(2.7)b	46.3(0.6)b	50.7(0.6)ab	0.004
*G. spiculosa*	65.6(1.5)a	49.5(1.8)b	48.5(0.6)b	48.8(1.0)b	<0.001

Note: Data in parentheses are the standard errors of means (*n* = 3). Different lowercase letters in the same rows indicated significant differences among the four treatments.

**Table 3:** Effect of P addition on plant nutrient resorption at the community level

	Control	Low P	Moderate P	High P	*P*-values
N resorption proficiency (mg g^−1^)	3.95(0.15)c	5.32(0.22)b	5.89(0.22)ab	6.14(0.11)a	<0.001
P resorption proficiency (mg g^−1^)	0.71(0.01)c	1.36(0.02)b	1.43(0.03)ab	1.52(0.02)a	<0.001
N resorption efficiency (%)	52.3(1.5)	53.5(0.9)	52.5(0.8)	56.6(1.9)	0.169
P resorption efficiency (%)	60.6(0.6)a	48.9(2.1)b	47.7(0.6)b	49.1(0.9)b	<0.001
N:P resorption ratio	0.86(0.03)b	1.10(0.05)a	1.10(0.01)a	1.15(0.02)a	0.001

Note: Data in parentheses are the standard errors of means (*n* = 3). Different lowercase letters in the same rows indicated significant differences among the four treatments.
