# Supplementary material for: Responses of plant nutrient resorption to phosphorus addition in freshwater marsh of Northeast China
Source: Sci Rep. 2015 Jan 29;5:8097. doi: 10.1038/srep08097 (PMC4309954; doi:10.1038/srep08097)
Supplement: Supplementary Information — Responses of plant nutrient resorption to phosphorus addition in freshwater marsh of Northeast China [file srep08097-s1.doc]

Responses of plant nutrient resorption to phosphorus addition in freshwater marsh of Northeast China

Rong Mao^a^,*, De-Hui Zeng^b^, Xin-Hou Zhang^a^, Chang-Chun Song^a^,*

^a^ Key Laboratory of Wetland Ecology and Environment, Northeast Institute of Geography and Agroecology, Chinese Academy of Sciences, Changchun 130102, China

^b^ State Key Laboratory of Forest and Soil Ecology, Institute of Applied Ecology, Chinese Academy of Sciences, Shenyang 110016, China

**Table S1** Effect of P addition on mature plant organ nutrient concentration in a freshwater marsh, Northeast China

| Treatment | N concentration (mg g^-1^) | | | | P concentration (mg g^-1^) | | | |
| --- | --- | --- | --- | --- | --- | --- | --- | --- |
|  | *D.* *angustifolia* | | *G.* *spiculosa* | | *D.* *angustifolia* | | *G.* *spiculosa* | |
|  | Leaf | Stem | Leaf | Stem | Leaf | Stem | Leaf | Stem |
| Control | 11.94(0.46)b | 2.32(0.14)b | 13.84(0.08)b | 3.19(0.29)b | 1.56(0.09)c | 1.10(0.04)b | 1.82(0.15)b | 1.33(0.06)b |
| Low P | 15.35(0.32)a | 3.99(0.25)a | 15.23(0.13)a | 4.81(0.17)a | 2.40(0.14)b | 1.42(0.07)ab | 2.49(0.09)a | 1.95(0.08)a |
| Moderate P | 16.37(0.68)a | 3.45(0.18)ab | 15.97(0.27)a | 5.08(0.10)a | 2.44(0.03)b | 1.36(0.03)ab | 2.57(0.08)a | 1.91(0.04)a |
| High P | 17.17(0.19)a | 4.33(0.54)a | 16.02(0.22)a | 5.35(0.51)a | 2.96(0.11)a | 1.64(0.12)a | 2.46(0.08)a | 2.06(0.05)a |

Note: Values in parentheses are the standard errors of the means (*n*=3). Different lowercase letters in the same column indicated significant differences among the four treatments

**Table S2** Results (*P*-values) of three-way ANOVAs on the effects of P addition (P), species (S), plant organs (O) and their interactions on nutrient resorption parameters

|  | Inorganic P | Organic P | N resorption proficiency | P resorption proficiency | N resorption efficiency | P resorption efficiency | N:P resorption ratio |
| --- | --- | --- | --- | --- | --- | --- | --- |
| P | <0.001 | <0.001 | <0.001 | <0.001 | 0.652 | <0.001 | <0.001 |
| S | 0.001 | <0.001 | 0.177 | <0.001 | <0.001 | 0.272 | <0.001 |
| O | <0.001 | <0.001 | <0.001 | <0.001 | <0.001 | <0.001 | <0.001 |
| P×S | <0.001 | 0.009 | 0.157 | 0.003 | 0.622 | 0.001 | 0.030 |
| P×O | 0.098 | 0.016 | 0.013 | 0.001 | 0.289 | 0.148 | 0.353 |
| S×O | 0.744 | <0.001 | 0.021 | <0.001 | <0.001 | 0.728 | <0.001 |
| P×S×O | 0.012 | 0.024 | 0.337 | <0.001 | 0.219 | 0.005 | 0.028 |
